# Cascading trait-mediated interactions induced by ant pheromones

**DOI:** 10.1002/ece3.322

**Published:** 2012-07-27

**Authors:** Hsun-Yi Hsieh, Heidi Liere, Estelí J Soto, Ivette Perfecto

**Affiliations:** 1 School of Natural Resources and Environment, University of MichiganAnn Arbor, Michigan, 48109; 2 Department of Entomology, University of WisconsinMadison, Wisconsin, 53706; 3 Departmento de Agroecología, Universidad Autónoma de ChapingoTexcoco, México

**Keywords:** Alarm signaling, ant–hemipteran mutualism, coffee agroecosystem, complex ecological network, predator avoidance, TMII

## Abstract

Trait-mediated indirect interactions (TMII) can be as important as density-mediated indirect interactions. Here, we provide evidence for a novel trait-mediated cascade (where one TMII affects another TMII) and demonstrate that the mechanism consists of a predator eavesdropping on chemical signaling. Ants protect scale insects from predation by adult coccinellid beetles – the first TMII. However, parasitic phorid flies reduce ant foraging activity by 50% – the second TMII, providing a window of opportunity for female beetles to oviposit in high-quality microsites. Beetle larvae are protected from ant predation and benefit from living in patches with high scale densities. We demonstrate that female beetles can detect pheromones released by the ant when attacked by phorids, and that only females, and especially gravid females, are attracted to the ant pheromone. As ants reduce their movement when under attack by phorids, we conclude that phorids facilitate beetle oviposition, thus producing the TMII cascade.

## Introduction

Long appreciated by traditional naturalists, the idea of a trophic cascade gained popularity in the ecological literature through debates that emerged from Hairston, Smith, and Slobodkin's publication of the famous “green world hypothesis” (Hairston et al. 1960). The enormous literature that emerged from that article cemented in the consciousness of most ecologists the fact that ecological complexity pervaded ecosystems (e.g. Terborgh and Estes 2010). Also long appreciated, at least since Darwin, are the indirect effects on morphologies (and/or physiologies and/or behaviors, etc.…) so predominant in nature. In modern literature, these special indirect effects that involve some sort of “trait” of the organism are distinguished from those that affect only the density or biomass of the organism and are thus termed trait-mediated indirect interactions (TMII). It has been shown that, at least in some circumstances, the TMIIs can be so strong as to overwhelm any density-mediated effects (Abrams 1995; Werner and Peacor 2003).

Not yet appreciated to any great extent is another formulation that is likely to be familiar to naturalists, although thus far gaining little more than tacit appreciation (e.g. Bolker et al. 2003). For example, a parasitized mouse may have its food-searching ability (a trait) dramatically reduced by an ectoparasite, but the presence of a particular type of vegetation exposes the mice to less parasite attack than in its absence. The vegetation affects the ability (trait) of the parasite to affect the ability (trait) of the mouse to eat – a cascade of trait-mediated effects (species 1 affects the ability of species 2 to affect the ability of species 3 to do something). As with trophic cascades, one's imagination can create long links from simple natural history storytelling. Although this sort of linkage between TMII units has been discussed in the literature (Schmitz et al. 2004; Ohgushi 2005; Kessler and Halitschke 2007; Poelman et al. 2008; Utsumi et al. 2010), to our knowledge, the specific structure has not been documented experimentally for any terrestrial system other than the one here described (Liere and Larsen 2010). We, here, report on the complex chemical signaling that constitutes the mechanisms driving the TMII cascade.

Many TMII are induced by chemical volatiles, such as plant volatiles released in response to herbivory (reviewed by Agrawal 2005; Kessler and Halitschke 2007; Dicke et al. 2009) or predator chemicals that can be detected by the prey and trigger a behavioral change (Rothley et al. 1997; Schmitz et al. 1997, 2004; Relyea 2003; Schmidt-Entling and Siegenthaler 2009; Barbasch and Bernard 2011). Species interactions facilitated by chemical volatiles are common in aquatic and terrestrial insect communities (Bolker et al. 2003; Werner and Peacor 2003; Cardé and Millar 2004; Schmitz et al. 2004), and direct vertical hierarchies have been well documented, whether plant–herbivore, or host–prey interactions (Cardé and Millar 2004). Tri-trophic interactions involving chemicals are also well known in the case of predators and parasitoids that use plant volatiles to find their herbivore host (Vet and Dicke 1992; Ode 2006). However, the exploitation of insect communication systems by other species is less well documented – examples include the use of bark beetle aggregation pheromones by their predators (Wood 1982), the use of a sex pheromone emitted by male stink bugs by their tachinid fly parasitoids (Aldrich et al. 2007), and the use of moth pheromones by egg parasitoids (Fatouros et al. 2008). Beside the well-known cases of chemical camouflage and mimicry (Hölldobler and Wilson 1990; Dettner and Liepert 1994), surprisingly little is known of insects that exploit ant chemical communication systems, even though ant pheromones are especially reliable sources of information (Hölldobler and Wilson 1990). Reported cases include two ant-eating spiders that use ant alarm pheromones to find their prey (Allan et al. 1996; Clark et al. 2000), phorid parasitoids that use ant alarm or trail pheromones to find their host (Feener et al. 1996; Morrison 1999; Chen and Fadamiro 2007; Mathis et al. 2011; Mathis and Philpott 2012), and two coccinellid beetles, one that uses ant alarm pheromones to find aphids being tended by the ants (Godeau et al. 2003) and the other using ant pheromones to avoid oviposition sites where ants are the most active (Oliver et al. 2008). However, more complex cascading trait-mediated interactions that are facilitated by ant pheromones related to the presence of a third species and have direct consequences for population and community-wide dynamics have not been previously reported.

Ant–hemipteran interactions are among the most ubiquitous mutualisms in terrestrial ecosystems (Buckley 1987). By protecting hemipterans from their predators and parasitoids, ants have access to a reliable and abundant source of energy and nutrients (Buckley 1987), an example of a trait-mediated interaction as ants do not prey directly on the natural enemies, but rather harass them, thus reducing their access to the hemipterans.

Here, we experimentally demonstrate a cascade of trait-mediated indirect interactions involving two TMII units: (1) an ant–hemipteran mutualism unit, where the ants interfere with the ability of predators to attack scale insects, and (2) a phorid-ant-hemipteran unit where the phorids reduce the foraging activity of the ants, thus reducing their ability to interfere with the predator of the hemipteran mutualist. We also demonstrate that the linkage between the TMII units is mediated by semiochemicals, more specifically ant pheromones that are used for alerting nest mates about the presence of parasitoids, thus disrupting the ant's ability to interfere with the behavior of the predator, especially with the ability of the predator to engage in oviposition behavior.

### The study system: the first TMII unit

**Figure 1:**
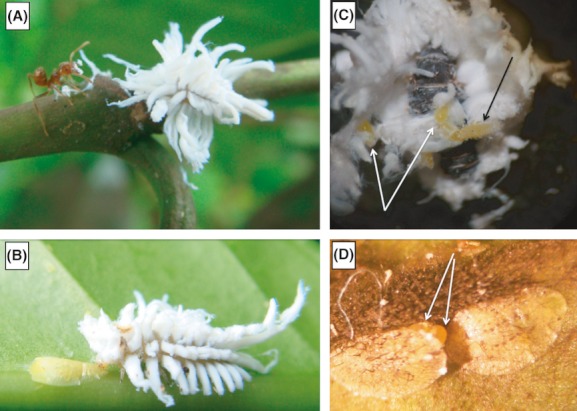
Photos of *Azya orbigera*: (A) The ant, *Azteca instabilis*, attacking *A. orbigera*, but getting its mandibles filled with the sticky waxy filaments that cover the body of the larvae of *A. orbigera*; (B) *A. orbigera* eating a *Coccus viridis*; (C) Eggs (white arrows) and first instar larvae (black arrow) of *A. orbigera* on an old pupal case of the same species; (D) *A. orbigera* eggs (white arrows) hidden under a scale insect.

In a typical ant–hemipteran association, the arboreally nesting ant, *Azteca instabilis*, protects a significant pest of coffee, the green scale, *Coccus viridis*, against most natural enemies (Vandermeer and Perfecto 2006). Consequently, high densities of green scales can only be found in association with *Azteca* ants. However, the predatory coccinellid beetle, *Azya orbigera*, has evolved a capacity to exploit this mutualism (Liere and Perfecto 2008). The larval form is covered with waxy filaments that effectively protect it from ant attacks (Fig. 1A), allowing it to live in areas with a high density of the green scale, its main prey (Fig. 1B) (Perfecto and Vandermeer 2008). Furthermore, by scaring away scale parasitoids, the ants inadvertently also scare away parasitoids of the beetle larva, thus providing it with enemy-free space (Liere and Perfecto 2008). On the other hand, adult beetles are harassed and could be killed by the ants (see Video S1). Both larvae and adults of the beetle are significantly more abundant on coffee plants around *Azteca* ant nests than in areas without ants, (see Table S1), suggesting that female beetles are ovipositing on ant-tended plants despite the risk of attacks or egg predation. As ants remove almost all beetle eggs laid bare on ant-tended plants (I. Perfecto, pers. obs.), female beetles must hide their eggs to protect them against ant predation. We have encountered coccinellid beetle eggs on old *A. orbigera* pupal cases that still have the waxy filaments (Fig. 1C) and under scale insects (Fig. 1D), suggesting that adult *A. orbigera* females are effectively searching out safe microsites for their eggs within ant patrolled plants. The ant-hemipteran mutualism is a well-established trait-mediated indirect interaction unit. However, the presence of coccinellid eggs under scale insects and the high density of coccinellid larvae and adults on plants with *Azteca* ants (Table S1) suggest that something is interfering with this TMII unit.

### The study system: the second TMII unit

A second TMII unit provides clues as to what could be interfering with the ant–hemipteran TMII unit. The phorid fly, *Pseudacteon laciniosus* (and possibly *P. planidorsalis* and *P. pseudocercus*. Brown and Philpott 2012) is a parasitoid of *A. instabilis* (Philpott et al. 2004). However, as with many other phorid parasitoids of ants, its main effect on the ant is a trait-mediated effect through a reduction of the ant foraging activity (Philpott et al. 2004, 2009; Philpott 2005; Hsieh and Perfecto 2012). Previous work demonstrated that these phorids are attracted to an alarm-defense pheromone produced by *A. instabilis*, and also that movement of the ants must be detected by the fly at close range for the latter to oviposit (Mathis et al. 2011). As a consequence of this requirement, when phorids arrive, the ants take on a motionless catatonic state (see Video S2). As this behavior becomes generalized among all ants in the vicinity after a phorid attack, it is evident that a phorid-alert pheromone is released to warn nearby workers that a phorid is in the vicinity. The result of this behavior is that overall activity of the ants declines by at least 50% in the presence of the phorids (Philpott 2005). This reduction in ant activity is sufficiently large to provide the coccinellid beetles with an effective temporal refuge from the ants (Liere and Larsen 2010). The effect of the phorids can last up to 2 h (Philpott et al. 2004), possibly providing the female beetles with enough time to find high-quality and secure oviposition sites.

The question then is, how do the beetles find these high-quality patches, and can the beetles take advantage of the low ant activity (when the phorids are present) by being able to detect the phorid-alert pheromone?

Here, we test the hypothesis that the coccinellid beetle, *A. orbigera*, is able to detect volatile pheromones from the ants and, more importantly, that the coccinellid beetles have the ability to detect the unique alarm pheromone (or pheromones) released by ants specifically when under attack by phorids, therefore taking advantage of a window of opportunity to search out safe sites for oviposition.

## Materials and Methods

To test the hypothesis that beetles are able to detect volatile pheromones released by ants, we employed a standard olfactometer (Pettersson et al. 1998). We used various chemical attractants of ants on female and male coccinellid beetles, and with mated and unmated females. We also conducted a beetle oviposition experiment, manipulating the presence of phorid flies to determine if the presence of phorids indeed produced a TMII cascade that facilitates beetle oviposition.

Beetles, ants, and phorid flies used to conduct the studies were collected from an organic coffee plantation in the southern part of the state of Chiapas, Mexico (15°10′N, 92°20′W) or reared in the laboratory after field collections from the same site. Five colonies of *A. instabilis* were collected from the field and maintained in the laboratory for the duration of the studies. Phorid flies were collected from the field minutes before they were used in the experiments. Individuals of *A. orbigera*, the coccinellid beetle, used in the first sets of olfactometer studies were collected directly from the field, kept in the laboratory, and fed with *C. viridis*. The individual beetles used to determine attraction of ant pheromones to mated and unmated female beetles and for the oviposition experiment were reared in the laboratory from larvae collected in the field.

### Olfactometer bioassays

To investigate whether the coccinellid beetle, *A. orbigera*, was able to detect and be attracted to pheromones from *A. instabilis*, we conducted behavioral assays in a two-arm olfactometer, modified from the four-armed Perplex olfactometer (Pettersson et al. 1998). The apparatus consisted of a central arena 6 × 6 cm with two conical extended arms of 7 cm, to which odors could be introduced from source chambers connected with tubing via 4 mm holes at each at the end of each arm. Airflow in the arena was created by connecting a tube to the center of the arena and attaching it to an air pump, effectively creating two odor environments, a control, and a treatment. The olfactometer was divided into three zones, a neutral zone, consisting of 2 cm in the center of the arena, and the stimulus and control zones, extending to the right and left of the neutral zone and both consisting of 9 cm, separated into six units of 1.5 cm each. At the start of each trial, one beetle was placed in the neutral zone and after a short acclimatization period, the position of the beetle was recorded every 20 sec for 5 min (15 recordings per trial). The position of the treatment was altered every few trials. After each trial, the arena was cleaned with 70% ethanol.

To test beetles' response to the general odor of ants, 20 ants were placed in one of the source chambers and the other left empty. We waited for at least 10 min to let the ants calm down before placing the beetle in the arena and running the assay. To determine beetle attraction to ant pheromones, 20 ants were crushed and placed in one of the source chambers and the other left empty. Crushing the ants liberates all the pheromones produced by ants and is a method frequently used in these types of bioassays (Brown and Feener 1991; Francis et al. 2004; Mathis et al. 2011). Finally, to test for the attraction of *A. orbigera* to *A. instabilis* while being attacked by phorid parasitoids, we collected five colonies of *A. instabilis* in carton nests and divided each of them into equal halves. Then we placed the two equal parts of each colony in two transparent 60 × 60 × 60 cm insect-rearing cages (Bug Dorm-2 Insect Tent; Bug Dorm Store, Taichung, Taiwan). We connected the cages to the two-arm olfactometer and waited for at least 10 min until the ants calmed down. We then released three to five freshly collected phorid flies into one of the tents and placed one adult beetle in the neutral zone of the olfactometer. When at least one phorid fly started attacking the ants, we started recording the location of the beetle. We conducted these trials with both male and female beetles.

To test the difference between mated and unmated females, we collected larvae and pupae of the beetle from the field and reared them to adults. Some were placed in containers with males and kept there until copulation was observed. The assays to examine differences in the response of mated and unmated females beetles were conducted with unmated females and mated females. We also conducted trials with females that had mated at different times.

To determine the source of the pheromone or pheromones that could attract coccinellid beetles, we conducted additional trials using various body parts of the ants. Both males and female beetles were tested for head, thorax, dorsal part of abdomen and ventral part of abdomen.

Finally, to determine if the adults of *A. orbigera* used olfactory cues to locate *C. viridis*, we used coffee leaves with *C. viridis* in one chamber and enclosed equal numbers of coffee leaves without *C. viridis* in the other chamber.

### Oviposition experiment

To determine if gravid females *A. orbigera* actually use the chemical cues of the ants when phorid flies are attacking them, we conducted an oviposition experiment. Female *A. orbigera* were collected from the field, collectively mated, placed in individual containers and daily satiated with scale insects in the lab. When oviposition of a female beetle was observed, the female beetle was used in the experiment. When at least three female beetles were ovipositing, we shuffled individual gravid beetles and randomly assigned them into one of three treatments: (1) no ants/no phorids, (2) ants/no phorids, and (3) ants + phorids. The chambers where the experiment was conducted consisted of containers of 1 L containing a coffee branch with four to six coffee leaves infected with scale insects. The ant/no phorid treatment contained 40 workers of *A. instabilis* and the ants + phorids treatment contained 40 workers of *A. instabilis* plus two to three phorid flies. After 24 h, the coffee branch was removed from the experimental chamber and placed under a dissecting microscope, where it was carefully examined for eggs of *A. orbigera*. The three individual gravid beetles were returned to the beetle pool with other individuals. For the next trial all three beetles were again randomly selected and assigned to treatments. The experiment was replicated nine times.

### Statistical analyses

Beetle preferences were determined by directly calculating the probability of finding something other than a 50% response with a binomial distribution using Excel binomial distribution function (*P* = 1−BINOMDIST [number of beetles that choose treatment, total number of trials, TRUE]). For each trial, we added all recordings for the control, the treatment, and the neutral (no-response) zones, and categorized the trial based on the zone that had the higher number of recordings (control or treatment). Trials categorized as “no-response” were eliminated from the analyses. To investigate the relationship between days after copulation and level of attraction to ant pheromones, a simple linear regression analysis was used. In this particular case, instead of using the categories (control or treatment), we calculated a strength index by averaging the number of the position of the beetle at each recording period (from 1 to 6, with 1 being a weak response and 6 being a strong response). To test whether ants deter female beetles from ovipositing and whether phorid parasitoids facilitate oviposition, a Wilcoxon rank-sum test was conducted.

## Results

**Figure 2:**
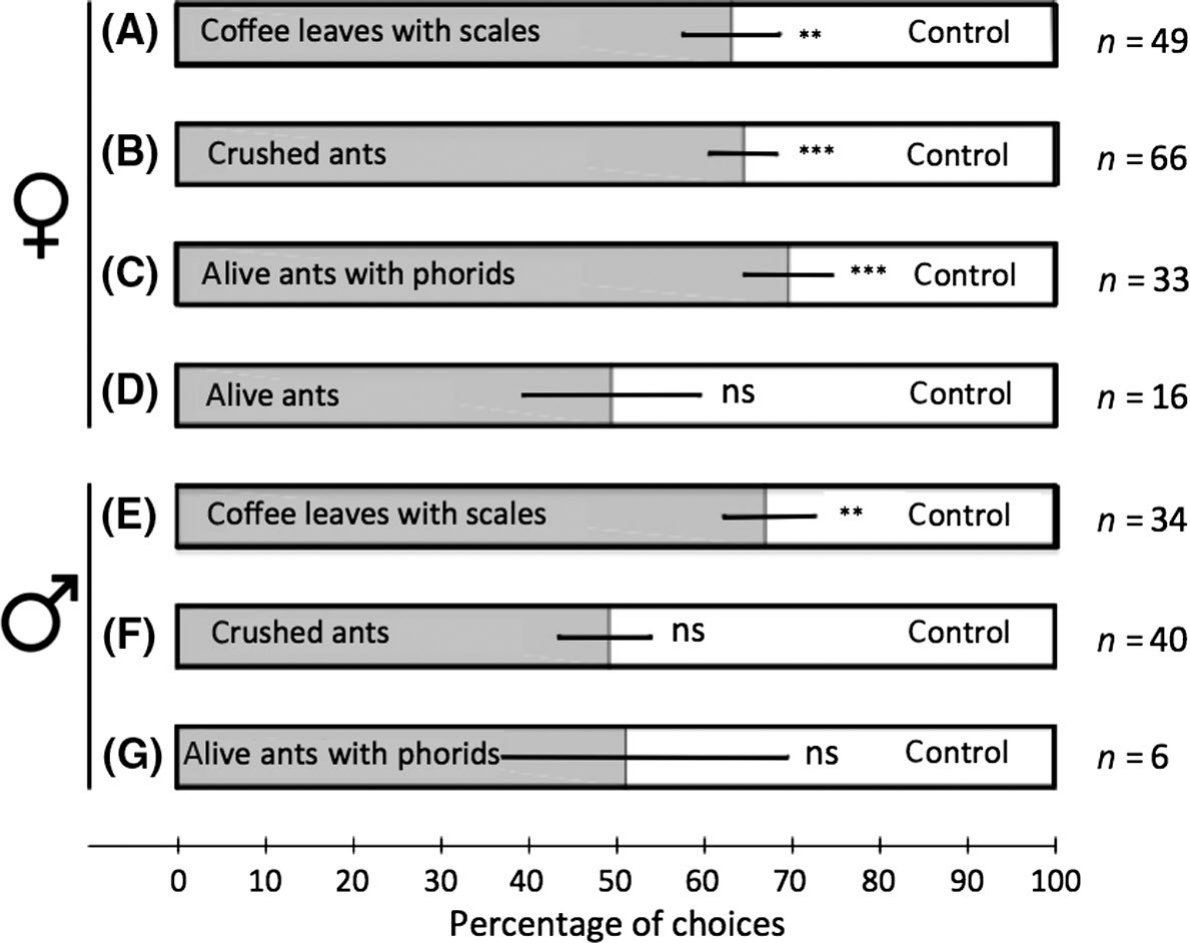
Percentage of female and male beetles choosing control versus treatment. Asterisks indicate statistical significance based on binomial distribution; **P = < 0.01; ***P = < 0.001. Error bars based on standard error of the mean.

Both females and males of the coccinellid beetle, *A. orbigera*, are attracted to the green coffee scale (Fig. 2; see also Fig. S1). However, only females showed any response to odors released by ants (Fig. 2). While females showed no response to live ants that were not being attacked by phorids or that were not alarmed for other reasons, they did show a positive response to crushed ants and to live ants that were being attacked by phorid flies (Fig. 2B and C).

**Figure 3:**
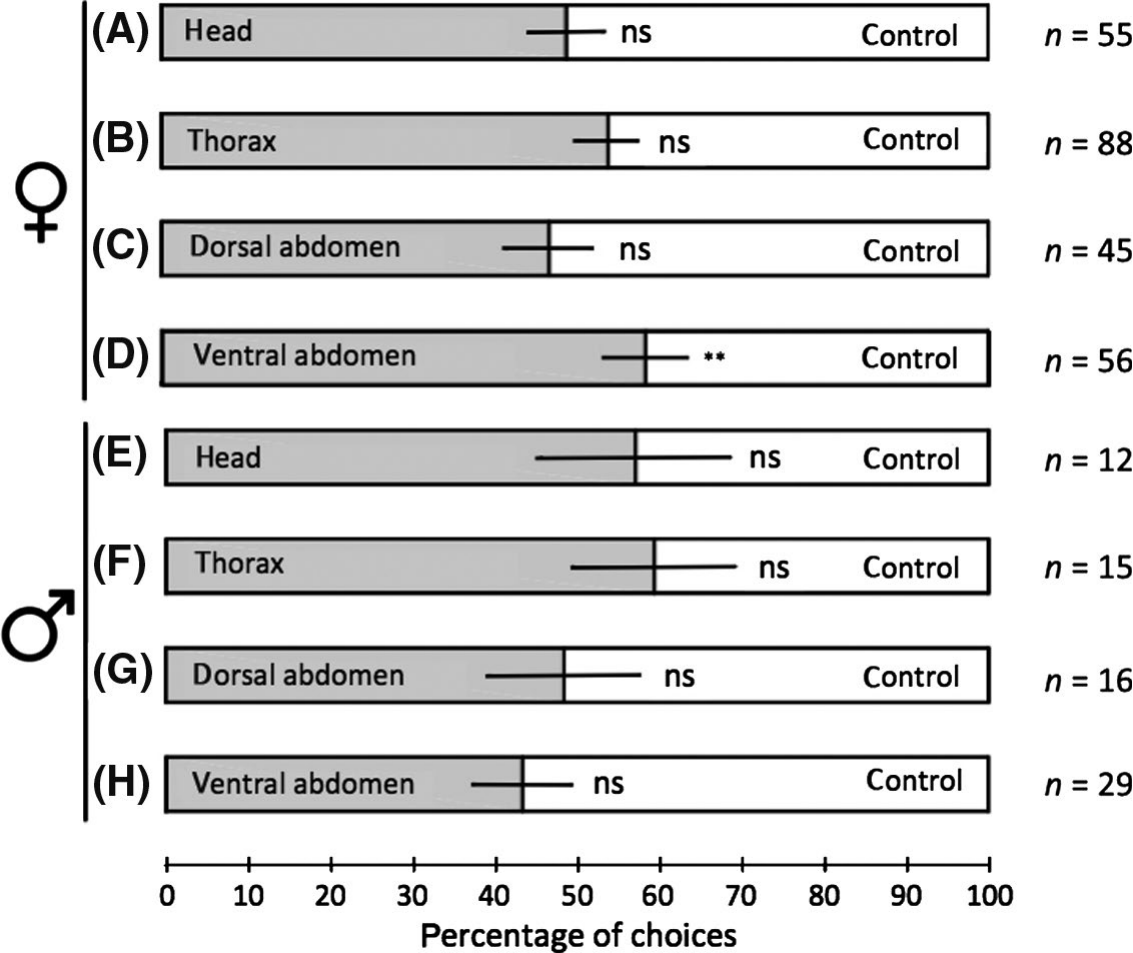
Percentage of female and male beetles choosing control versus treatment (various ant body parts). Asterisks indicate statistical significance based on binomial distribution; **P = < 0.01; ***P = < 0.001. Error bars based on standard error of the mean.

Through a separate series of two-arm olfactometer assays (testing heads, thoraxes, ventral part of abdomens, and dorsal part of abdomens), we were able to determine that the phorid-alert pheromone is produced in the ventral part of the abdomen, most likely in the Pavan or Dufour's gland (Fig. 3), distinct from the general alarm pheromone that attracts the phorids and that is produced in the pygidial gland, located on the dorsal side of the abdomen (Mathis et al. 2011). These assays also confirmed that only female beetles are attracted to pheromones produced by ants (Fig. 3D).

**Figure 4:**
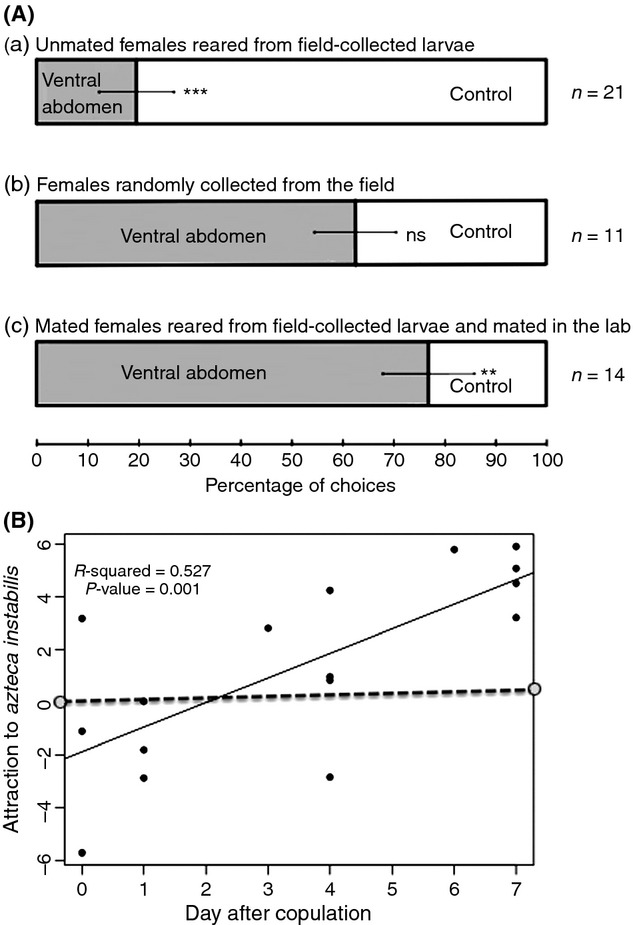
Response of mated and unmated female *Azya orbigera* to ant pheromones. (A) Percentage of unmated, mated, or field-collected female beetles choosing control versus odor from the ventral abdomen of ants. Asterisks indicate statistical significance based on binomial distribution; **P = < 0.01; ***P = < 0.001. Error bars based on standard error of the mean. (B) Relationship between days after copulation of female *A. orbigera* and attraction to ants. The dashed horizontal line divides beetles' positive and negative responses to ant pheromones, with numbers representing the intensity index (O = no response; 6 = strong response). *P*-values based on a simple linear regression between days after copulation and level of attraction to ant odor.

Testing mated and unmated females demonstrated that the attraction to ant pheromones is manifested only after mating, and that before mating, female beetles have an aversion to ant pheromones (Fig. 4A). Female beetles collected from the field showed an intermediate level of attraction to ant pheromones between that of unmated and mated females (Fig 4A). Furthermore, the attraction to ant pheromones continues to increase for at least 7 days after copulation (Fig. 4B).

**Figure 5:**
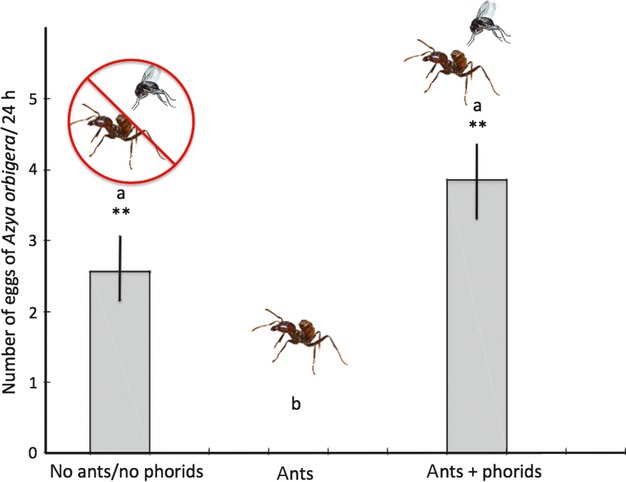
Number of eggs oviposit by *Azya orbigera* adult in insect cages with branches of coffee with scale insects, but no ants or phorid flies (no ants/no phorids), with branches of coffee with scale insects and ants, but no phorid flies (ants), and with branches of coffee with scale insects, ants and phorid flies (ants + phorids). Statistical test based on Wilcoxon rank-sum test (treatment comparisons: ants + phorids versus ants, *P*-value = 0.015; phorids versus no ants/no phorids, *P*-value = 0.014; no ants/no phorids versus ants + phorids, *P*-value = 0.87). Mean eggs within 24 h + SEM: 2.56 ± 0.40 (neither), 0 ± 0.00 (ants); 3.86 ± 0.55 (ants + phorids).

Results for the oviposition experiment show that the average number of eggs oviposited by *A. orbigera* on the no ant/no phorid treatment is not significantly different from the average number of eggs oviposited on the ants + phorids treatment (Fig. 5). It also shows that both of these treatments, on the average, had higher eggs oviposited than the ant/no phorid treatment (Fig. 5). These results support the hypothesis that ants deter female beetles from ovipositing on plants where they tend scales, and that female beetles used the phorid-alert pheromone (or pheromones) released by *A. instabilis* to find a window of opportunity to oviposit (Fig. 5).

## Discussion

This study demonstrates that the coccinellid beetle, *A. orbigera*, is able to detect pheromones released by the ant *A. instabilis*. More interestingly, female beetles are attracted specifically to the phorid alarm pheromone released by the ants when under attack by *Pseudacteon* phorid flies. Furthermore, only gravid female beetles showed an attraction to the ant pheromones and this attraction increased with the number of days after copulation. Further evidence that female beetles are attracted to the ants only when they are being attacked by phorids comes from the lack of a significant response to live, undisturbed ants (Fig. 2). These results also explain the large variance encountered when olfactometer assays are conducted without discriminating among female beetles' gravid status (i.e. beetles collected from the field).

This study also provides strong evidence that the female beetles use the phorid-alert pheromone released by the ants to find windows of opportunity to oviposit in high-quality sites where the larvae will have sufficient food (scale insects) and be protected from parasitoids (Liere and Perfecto 2008). We also showed that both male and female beetles are attracted to chemicals released by scale insects or coffee volatiles induced by herbivory (see online supplementary materials and Fig. S1).

Given these results, it is likely that both male and female beetles find patches of high concentrations of scales through chemical compounds found either in the scale insects themselves or from volatiles emitted by coffee plants when being fed on by scales (see Fig. S1). As beetle larvae have dramatically restricted movements and are attacked by several parasitoids (Liere and Perfecto 2008), there is clear pressure for female beetles to oviposit in ant-tended areas, where high prey density and low risk of parasite attack are secured. However, the aggressive behavior of ants renders female beetles incapable of ovipositing in these high-quality areas (Fig. 5). Here, we demonstrate that female beetles can avoid this problem by being able to detect the phorid-alert pheromones released by *Azteca* ants. This ability allows beetles to take advantage of the low ant-activity periods to search for sites where their eggs can be hidden and protected against ant predation after ants resume their normal activity levels.

**Figure 6:**
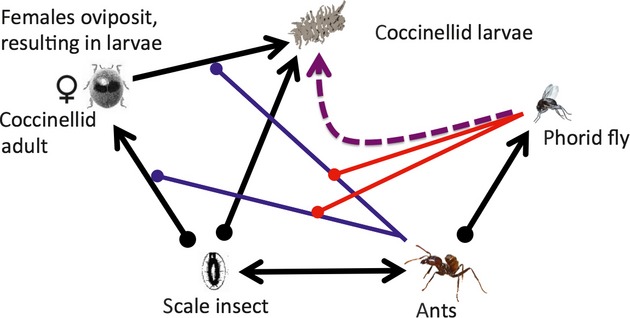
Diagrammatic representation of the cascading trait-mediated indirect interactions between the phorid flies, *Pseudacteon* spp., and the coccinellid beetle, *Azya orbigera*. Arrows represent positive effects and solid circles represent negative effects. Black solid lines represent direct interactions, blue solid lines represent first-level trait-mediated indirect interactions, red solid lines represent second-level trait-mediated indirect interactions, and dashed purple line represents the resulting cascading trait-mediated indirect interaction between the phorid flies and the coccinellid beetle larva.

More generally, this system is an example of cascading trait-mediated indirect interactions resulting from the linkage between two TMII units: the ant-scale mutualism unit and the ant-phorid-scale unit. Furthermore, in this study, we were able to demonstrate that the linkage between these two TMII units is mediated by the ability of the beetle to eavesdrop on the chemical signaling of the ant. In other words, the ant pheromone initiates the cascading trait-mediated indirect interactions that result in the facilitation of the coccinellid beetle. The first TMII is the ant's interference with the ability of the female beetles to oviposit in sites with high scale abundance, due to the ants' mutualistic interaction with the scale insects. The second TMII is the phorid fly interference with the first TMII (i.e. with the ant's interference of beetle oviposition). These interactions are illustrated in Figure 6. Through those cascading trait-mediated indirect interactions (effects on effects on effects), the phorid fly indirectly facilitates the coccinellid beetles.

The facilitation that results from the linkage between these two TMII units can have important consequences at the ecosystem level. The scale, *C. viridis*, is reported to be an important pest of coffee causing considerable damage in some cases (Williams 1982). The coccinellid beetle, *A. orbigera*, is the main regulator of the scale insect at the landscape level (Perfecto and Vandermeer 2008; Vandermeer et al. 2010). Outside the patches of *Azteca*, the larvae of *A. orbigera* suffers a high percentage of parasitization by at least four different parasitoids and is unlikely to reach maturity and reproduce (Liere and Perfecto 2008; Vandermeer et al. 2010). Therefore, the *Azteca* patches represent refuges for the larvae of *A. orbigera*. Once the adults emerge, they fly away from the *Azteca* patches, thus escaping harassment by the ants. Mark-recapture experiments show that adult beetles move readily between *Azteca* patches (H. Liere, unpubl. data), suggesting that they control scale insects outside *Azteca* patches, which in the study area represent only about 3–5% of the coffee area (Perfecto and Vandermeer 2008; Vandermeer et al. 2008).

This is the first report of an insect exploiting the ant chemical communication system through such complex interactions. In other cases, parasitoids or predators were able to use a trail or an alarm pheromone to locate ants to attack them (Allan et al. 1996; Feener et al. 1996; Clark et al. 2000; Chen and Fadamiro 2007; Mathis et al. 2011), locate their hemipteran mutualists (Godeau et al. 2003), or avoid them altogether (Oliver et al. 2008). It is not difficult to imagine how these direct chemically mediated interactions evolved as ant pheromones are known to be reliable sources of information (Hölldobler and Wilson 1990). The evolution of such complex interactions as reported here is more perplexing, especially given that it occurs in an agroecosystem of a non-native species, coffee. *Coffea arabica*, the host plant of *C. viridis*, is not native to the Americas, having been introduced less than 300 years ago from Africa (Vega 2008). *Coccus viridis* is a pan-tropical phytophagous insect thought to have originated either in Ethiopia or Brazil (Zimmerman and Hardy 1948; Gill et al. 1977). The phorids, *Pseudacteon laciniosus*, and possible two other species were recently described as attacking only *A. instabilis* (Brown and Philpott 2012), therefore it is likely that they are restricted to the geographic range of *A. instabilis* in the Americas. Although the genus *Pseudacteon* has been collected from the Americas, Europe, Asia, and Australia, its center of diversification is reported to be Brazil (Porter and Pesquero 2001). Finally, *A. instabilis* is a Neotropical ant whose distribution ranges from Brazil to Mexico (Kempf 1972). Except for coffee, the host plant, the other species involved in this network are likely to have been interacting in natural systems for sufficient time to allow the evolution of such complexity.

Our study demonstrates that *A. orbigera*, the coccinellid beetle, has the ability to capitalize on the “phorid alert” pheromones released by the ant *A. instabilis* while under attack by phorid flies. Phorid attacks reduce ant foraging activity, thereby providing female coccinellid beetles with a window of opportunity to oviposit in sites with high scale density. This is an example of linkages between TMII units with potentially important ecosystem-level implications (Schmitz et al. 2004; Ohgushi 2005; Kessler and Halitschke 2007; Ohgushi et al. 2007; Poelman et al. 2008; Utsumi et al. 2010).

These chemically mediated indirect complex interactions can have practical implications for the management of coffee, as C. *viridis*, the scale insect, is a pest in coffee, the coccinellid beetle is an important predator of the scale insect and their effect on scales is clearly conditioned by the cascading TMII described here. Given that this system is found in a recently established agroecosystem, it is likely that components of the system (the TMII units) are quite common in natural ecosystems and that these sorts of TMII cascades could be more common than previously thought.

To our knowledge, ours is one of the few studies that document these cascading effects (the others being from the same study system; Liere and Larsen 2010; Pardee and Philpott 2011; Philpott et al. 2012), and the only one investigating the mechanism behind the cascading interactions. However, each pairwise interaction involved in this system is common in nature: (1) ant/hemipteran mutualisms are very prevalent and well documented (Buckley 1987), (2) phorid parasitoids frequently elicit trait-mediated effects (Hsieh and Perfecto 2012), (3) ants depend on fairly reliable pheromones for communication among nest mates (Hölldobler and Wilson 1990), and (4) many insect predators and parasitoids have been found to use other insect chemicals to find their prey/host (Wood 1982; Bolker et al. 2003; Werner and Peacor 2003; Cardé and Millar 2004; Schmitz et al. 2004; Aldrich et al. 2007; Chen and Fadamiro 2007). Furthermore, recent studies suggest that TMII do not occur in isolated units, but interact with other TMII units (Utsumi et al. 2010). Given the prevalence of these pairwise interactions and the documented linkages between TMII units, it is likely that the complex trait-mediated cascade of interactions documented here are common in nature, but due to the complexity of the interactions have not been documented in other systems.

Ecological research long ago transcended the limits of classical density-dependent interactions, and the literature is now replete with examples of chemical, behavioral, and morphological changes of organisms in response to challenges from other organisms, such as the threat of predation. Indeed, such trait-mediated indirect interactions significantly affect species coexistence, trophic length, and energy and material flows within ecosystems (Werner and Peacor 2003; van Veen et al. 2005, 2009; Kratina et al. 2010; Loreau 2010; Kefi et al. 2012). Less attention has been accorded to what we report herein, the possibility of cascades of trait-mediated effects. We suggest that such effects may be common in nature and their uncommon occurrence in the literature is a product of investigators failing to search for them in the first place. Current literature of simple trait-mediated interactions is in need of empirical support to bridge gaps generated by theoretical models (Bolker et al. 2003), while theoretical synthesis is expeditiously integrating non-feeding interactions into food webs (e. g. Bolker et al. 2003; Golubski and Abrams 2011; Kefi et al. 2012). However, we also emphasize that more complicated pathways, such as those described herein, may be paramount in affecting system dynamics and deserve more attention, both theoretical and empirical.

## Supporting Information

**Table S1.** Average number of coccinellid beetles found in coffee bushes in areas with and without *Azteca instabilis*. The numbers in parentheses are standard errors.

**Table S2.** Number of trial and *P*-values for binomial distribution tests for olfactometer trials.

**Figure S1.** Results of trials with a two-arm olfactometer for male and female *Azya orbigera* combined (*N* = 16, *P* = 0.20 direct binomial test).

**Video S1.** The ant, *Azteca instabilis*, following and harassing the coccinellid beetle, *Azya orbigera* on a coffee plant.

**Video S2.** The phorid, *Pseudacteon laciniosus*, hovering over a paralized *Azteca instabilis* ant.
