# Supplementary material for: Cascading trait-mediated interactions induced by ant pheromones
Source: Ecol Evol. 2012 Jul 27;2(9):2181–91. doi: 10.1002/ece3.322 (PMC3488669; doi:10.1002/ece3.322)
Supplement: Supplementary file 1 [file ece30002-2181-SD2.doc]

**On-line Supporting Information**

**1.** **The coccinellid beetle, *Azya orbigera*, is associated with the ant, *Azteca instabilis***

Larvae and adults of *Azya orbigera* are found at significantly higher densities within clusters of nests of *Azteca instabilis* than in areas with no ants. In a 45-hectare plot within an organic coffee plantation we mapped all shade trees and all *A. instabilis* nests (Vandermeer et al., 2008). The plot was divided into 128 subplots of 50X50 m. If no trees with *A. instabilis* nests were found within the plot, then the tree closest to the center of the plot was selected and the 5 closest coffee bushes were inspected for beetles and the number of *A. orbigera* larvae and adults were recorded. If the plot had trees with *A. instabilis* nests, then the tree with *A. instabilis* that was closest to the center of the plot was selected and the coffee bushes that were within a 3 meter radius from the center tree were inspected for beetle presence. The survey was conducted in the summer of 2006. The results of the survey are presented in table S1.

Table S1. Average number of coccinellid beetles found in coffee bushes in areas with and without *Azteca instabilis.* The numbers in parentheses are standard errors.

|  | With ants | Without ants | P |
| --- | --- | --- | --- |
| Adults | 5.76 (0.99) | 1.26 (0.21) | <0.000001 |
| Larvae | 3.8 (0.82) | 1.58 (0.32) | 0.005 |

**2. The coccinellid beetle*, Azya orbigera*, is attracted to volatiles of green coffee scales but not** **attracted to pierced coffee leaves without scales.**

Many plants are known to produce volatiles when damaged by herbivores (Paré and Tomlinson, 1999). We conducted olfactometry experiments using coffee leaves with scale insects and intact leaves and concluded that *A. orbigera* is attracted to leaves with scale insects (Fig. 2a,e). However, in order to determine whether the coccinellid beetles are attracted to volatiles from the scales or induced volatiles from the coffee leaves we conducted additional olfactometer experiments with intact and pierced leaves without scales. Although the production of volatiles can vary from insect damage to mechanical damage, the difference tends to be more in quantity than quality or in the proportion of the volatiles produced (Mithófer et al., 2005). Therefore, if the coccinellids are attracted to leaf volatiles rather than scale volatiles, we expect to find a difference between intact coffee leaves and leaves that have been pierced to mimic scale insect damage.

To conduct these tests, coffee leaves without green coffee scales were collected from the field and wrapped with wet cotton and parafilm within a few seconds of collection. Equal numbers of leaves of similar size were used for the treatment and the control. Twelve female and three male adults of *Azya orbigera*, one at a time, were used in the olfactometry experiment to test if the beetles were attracted to volatiles released from pierced coffee leaves as opposed to intact leaves. A direct binomial distribution test and a Mann-Whitney test showed that *A. orbigera* adults are not attracted to the volatiles released from pierced leaves (Fig. S1).


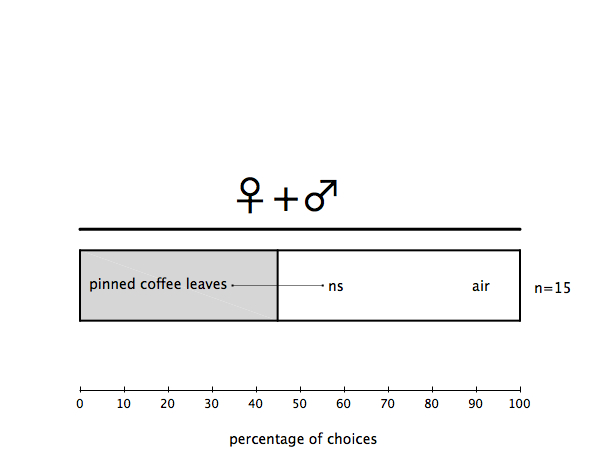


Figure S3. Results of trials with a two-arm olfactometer for male and female *Azya orbigera* combined (N=16, p=0.20 direct binomial test).

**3. Table S2**

Table S2. Number of trial and p values for binomial distribution tests for olfactometer trials.

|  |  | Number of trials | p value from Binomial Distribution | |  |
| --- | --- | --- | --- | --- | --- |
| Females | Scale insect | 49 | 0.004 |  | |
|  | Smashed dead ants | 66 | 0.000 |  | |
|  | Alive ants | 16 | 0.402 |  | |
|  | Phorid attacks | 33 | 0.000 |  | |
|  | Ant heads | 55 | 0.658 |  | |
|  | Ant thoraxes | 88 | 0.139 |  | |
|  | Ventral abdomens | 63 | 0.015 |  | |
|  | Dorsal abdomens | 45 | 0.674 |  | |
|  |  |  |  |  | |
| Males | Scale insect | 34 | 0.000 |  | |
|  | Crushed dead ants | 43 | 0.622 |  | |
|  | Alive ants | NA | NA |  | |
|  | Phorid attacks | 6 | 0.344 |  | |
|  | Ant heads | 12 | 0.133 |  | |
|  | Ant thoraxes | 17 | 0.402 |  | |
|  | Ventral abdomens | 29 | 0.907 |  | |
|  | Dorsal abdomens | 16 | 0.402 |  | |

**References**

Vandermeer, J., Perfecto & Philpott, S.M. Clusters of ant colonies and robust criticality in a tropical agroecosystem. *Nature* **451**, 457-459 (2008).

Mithófer, A., Wanner, G. & Boland, W. Effect of feeding *Spodoptera litoralis* on Lima bean leaves. II. Continuous mechanical wounding resembling insect feeding is sufficient to elicit herbivory-related volatile emission. *Plant Physiology* **137**, 1160-1168 (2005).

Paré, F.W. & Tumlinson, J. H. Plant volatiles as a defence against insect herbivores. *Plant Physiology* **121**, 325-332 (1999).
